# Disinfecting Wipes and Barrier Resistance of Protective Clothing

**DOI:** 10.1001/jamanetworkopen.2025.39307

**Published:** 2025-10-24

**Authors:** F. Selcen Kilinc-Balci, Patrick L. Yorio, Zafer Kahveci, Christian Coby

**Affiliations:** 1 National Personal Protective Technology Laboratory, National Institute for Occupational Safety and Health, US Centers for Disease Control and Prevention, Washington, DC; 2 Office of the Director, Human Resources Office, Office of the Chief Operating Officer, US Centers for Disease Control and Prevention, Atlanta, Georgia; 3 National Personal Protective Technology Laboratory, National Institute for Occupational Safety and Health, US Centers for Disease Control and Prevention, Pittsburgh, Pennsylvania; 4 National Personal Protective Technology Laboratory, National Institute for Occupational Safety and Health, US Centers for Disease Control and Prevention, Morgantown, West Virginia

## Abstract

This comparative effectiveness study examines the association between the use of quaternary ammonia–based wipes with and without alcohol and protective clothing performance.

## Introduction

Protective clothing (PC), including gowns and coveralls, is crucial personal protective equipment (PPE) for health care and laboratory workers (HCLWs) to prevent transmission of contaminated fluids. During public health emergencies, PPE shortages often lead to extended use, limited reuse,^1^ and decontamination with disinfecting wipes. Wipes are also used for localized cleaning during PPE removal or when changing PC is not feasible.^2^ The association between wipe use and PC liquid barrier resistance (LBR) remains unknown. This laboratory-based study examined associations between wipe use and PC performance to inform HCLW protection.

## Methods

This comparative effectiveness study was deemed research not involving human participants and exempt from institutional review board approval and informed consent and was conducted consistent with applicable federal law and Centers for Disease Control and Prevention policy. The study followed STROBE and ISPOR reporting guidelines.

We tested 9 PC models with various materials and constructions used by HCLWs, including gowns with and without American National Standards Institute/Association for the Advancement of Medical Instrumentation PB70 (hereafter referred to as AAMI) LBR^3^ claims (level 2-4 isolation and surgical gowns) and 2 coveralls with high water and blood resistance (eTable, eFigure, and eMethods in Supplement 1). Decontamination used quaternary ammonia–based wipes with alcohol (QAA) and without alcohol (QA). Ten samples per PC model were tested for LBR (impact penetration [IP]^4^ and hydrostatic resistance [HR]^5^ per AAMI) after wiping of the entire test area. Samples were conditioned before testing^6^ and tested after gentle wiping of the outer surface. For drying conditions, samples were wiped, dried, and tested. The LBR was analyzed separately for PC with and without AAMI claims based on wiping and drying conditions and fabric type.

Analysis of variance with Bonferroni-adjusted, post hoc, pairwise comparisons was conducted for each condition. Drying effects were assessed by comparing control (unwiped), wet, and dried fabrics using robust *t* tests (equal variances not assumed). Analyses were conducted from June 6 to July 30, 2021, using SPSS, version 23 (IBM Corp), with 2-sided significance set at *P* < .001.

## Results

Wiping was associated with a reduction in the HR of most fabrics, except laminated and poly-reinforced types, which typically have plastic-like surfaces, regardless of wipe type or drying status (Figure; Table), and may be attributed to low surface tension of the wipe fluids. The HR of microporous spunbond films (spunbond-film–spunbond-meltblown-spunbond [SMS] fabric) remained unchanged with QAA wiping but dropped after QA wiping (mean [SD], 222.4 [59.5] cm H_2_O; *P* < .001) and further after drying (mean [SD], 134.2 [17.3] cm H_2_O; *P* = .001). After QA wiping, IP was unchanged but declined after drying (mean [SD], 0.003 [0.005] g; *P* < .001). The association between IP and QAA and QA wiping was more evident in fabrics with lower HR, such as medium-weight SMS (mean [SD], 20.97 [1.11] and 21.58 [0.84] g, respectively) and heavy-weight SMS (mean [SD], 17.93 [6.2] and 21.52 [1.03] g, respectively) (*P* < .001). Drying generally did not restore HR, but higher HR was observed for coated (mean [SD], 194.7 [42.4] cm H_2_O) and noncoated flashspun (mean [SD], 77.0 [19.7] cm H_2_O) coveralls after QA wiping and drying (*P* < .001). Impact penetration was associated with drying after QAA wiping of coated flashspun (mean [SD], 0.002 [0.004] g; *P* < .001) and QA and QAA wiping of flashspun (mean [SD], 0.003 [0.005] and 0.068 [0.142] g, respectively; *P* = .001) fabric.

**Figure.**
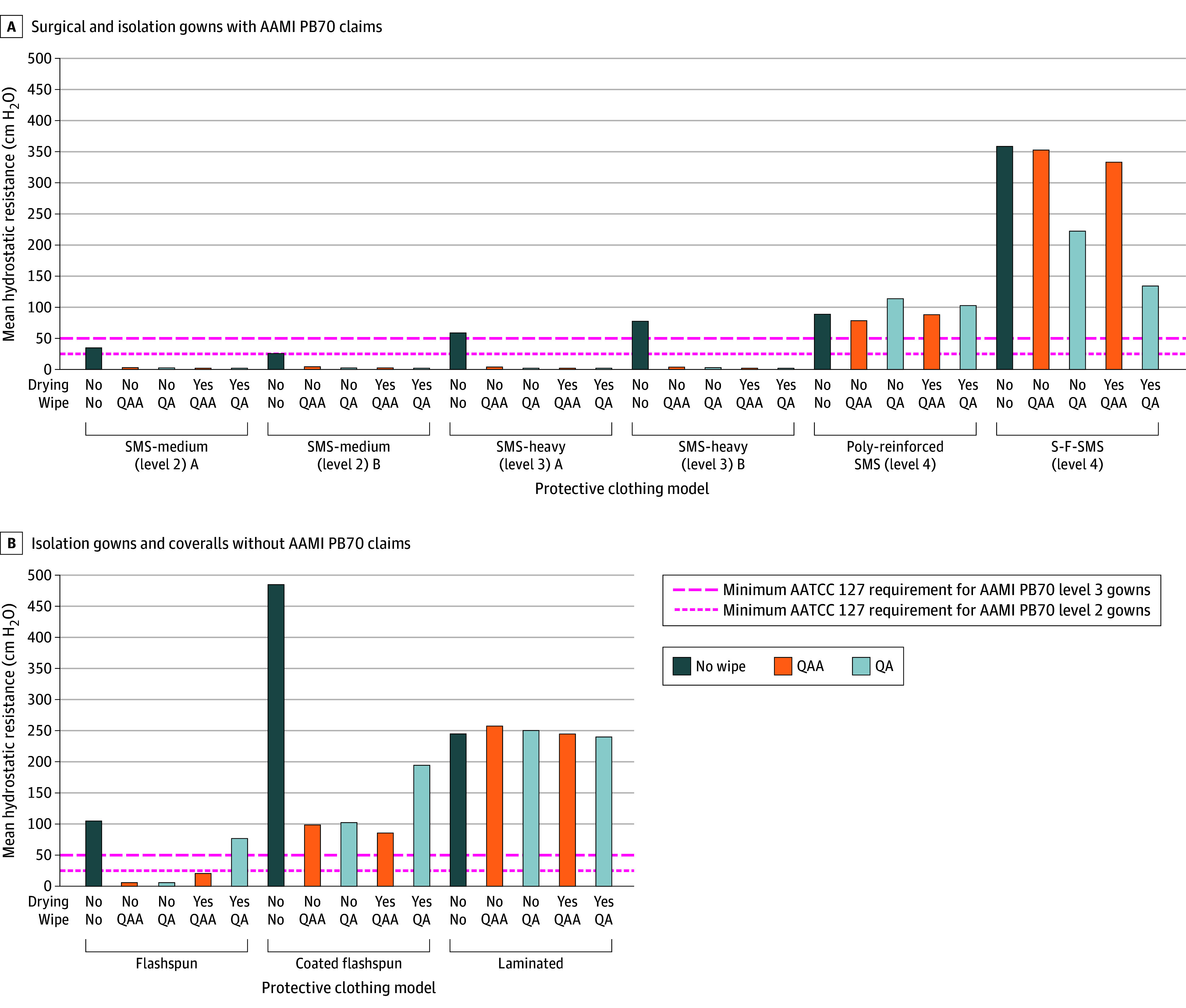
Association Between Disinfecting Wipe Application and the Hydrostatic Resistance of Gowns and Coveralls Drying condition yes indicates dried after wiping and no, not dried. AAMI indicates Association for the Advancement of Medical Instrumentation; AATCC, American Association of Textile Chemists and Colorists; QA, quaternary ammonia–based wipe without alcohol; QAA, quaternary ammonia–based wipe with alcohol; S-F-SMS, spunbond-film–spunbond-meltblown-spunbond.

**Table.** Descriptive Statistics for Impact Penetration and Hydrostatic Resistance of Gowns and Coveralls (Before Drying Condition)

Condition	Models, mean (SD)													
	AAMI PB70								Non-AAMI PB70					
	SMS medium weight (level 2)		SMS heavy weight (level 3)		Poly-reinforced SMS (level 4)		S-F-SMS (level 4)		Flashspun		Coated flashspun		Laminated	
	HR, cm H_2_O^a^	IP, g^b^	HR, cm H_2_O	IP, g	HR, cm H_2_O	IP, g	HR, cm H_2_O	IP, g	HR, cm H_2_O	IP, g	HR, cm H_2_O	IP, g	HR, cm H_2_O	IP, g
No wipe	30.2 (8.9)	5.13 (4.26)	68.3 (13.9)	0.06 (0.02)	88.8 (5.6)	0	358.7 (13.3)	0	105.1 (8.3)	0.02 (0.00)	485.0 (56.5)	0	245.0 (17.2)	0
QAA^c^	3.7 (0.9)	20.97 (1.11)	4.0 (1.1)	17.93 (6.2)	78.5 (40.7)	0	352.7 (19.5)	0.01 (0.01)	6.1 (1.5)	0.91 (0.59)	98.8 (10.4)	0.03 (0.01)	257.7 (16.9)	0
*P* value^d^	<.001	<.001	<.001	<.001	.80	>.99	>.99	>.99	<.001	>.99	<.001	>.99	.52	>.99
QA^e^	2.6 (0.6)	21.58 (0.84)	2.6 (0.5)	21.52 (1.03)	114.0 (37.7)	0	222.4 (59.5)	0.03 (0.01)	5.9 (0.7)	1.86 (1.26)	102.4 (11.7)	0	250.5 (7.2)	0
*P* value^d^	<.001	<.001	<.001	<.001	.02	>.99	<.001	>.99	<.001	>.99	<.001	>.99	>.99	>.99

Abbreviations: AAMI, Association for the Advancement of Medical Instrumentation; HR, hydrostatic resistance; IP, impact penetration; QA, quaternary ammonia–based wipe without alcohol; QAA, quaternary ammonia–based wipe with alcohol; S-F, spunbond-film; SMS, spunbond-meltblown-spunbond.

^a^ Hydrostatic resistance measured using American Association of Textile Chemists and Colorists (AATCC) 127 water resistance. Hydrostatic pressure test determines the ability of a material to resist water penetration under constant contact with increasing pressure.

^b^ Impact penetration measured using AATCC 42 water resistance. The IP test determines the ability of a material to resist water penetration under spray impact.

^c^ The QAA was the Super Sani-Cloth (PDI Healthcare) (bactericidal, tuberculocidal, virucidal). Active ingredients are n-alkyl dimethyl ethylbenzyl ammonium chlorides, n-alkyl dimethyl benzyl ammonium chloride, and isopropyl alcohol (0.25%, 0.25%, 55.00%, respectively); surface tension, 26.8 dyne/cm.

^d^ Comparisons between no wiping and wiping conditions for each wipe type, independent of drying. Values were obtained from analysis of variance with Bonferroni-adjusted, post hoc, pairwise comparisons. SPSS’s Bonferroni adjusted *P* value assumes a critical value of less than .05.

^e^ The QA was Clorox Disinfecting Wipes (The Clorox Company) (bactericidal, virucidal, fresh scent). Active ingredients are n-alkyl dimethyl benzyl ammonium chloride and n-alkyl dimenthyl ethylbenzyl ammonium chloride (0.184% and 0.184%, respectively); surface tension, 28.7 dyne/cm.

## Discussion

This comparative effectiveness study found that disinfecting wipes were associated with a decreased LBR across several PC models, underscoring the importance of avoiding this practice when maximum protection is needed. Consulting with PC manufacturers before using wipes may be helpful. Although most fabric types were covered, the study was limited by testing only a few PC and wipe models, and repeated wiping was not evaluated. Future investigation should include other PPE, including powered air-purifying respirator hoods and wipes containing bleach or hydrogen peroxide. These findings are especially important during pandemics, when wiping practices may increase due to PPE shortages.

## Notes

Supplement 1.
eMethods.
**eTable.** Protective Clothing Models **eFigure.** Microscopic Images of Fabrics at Low and High Magnification

Supplement 2.
Data Sharing Statement
