# Supplementary material for: Disinfecting Wipes and Barrier Resistance of Protective Clothing
Source: JAMA Netw Open. 2025 Oct 24;8(10):e2539307. doi: 10.1001/jamanetworkopen.2025.39307 (PMC12552923; doi:10.1001/jamanetworkopen.2025.39307)
Supplement: Supplement 1. — eMethods. eTable. Protective Clothing Models eFigure. Microscopic Images of Fabrics at Low and High Magnification [file jamanetwopen-e2539307-s001.pdf]

Supplemental Online Content

Kilinc-Balci FS, Yorio PL, Kahveci Z, Coby C. Disinfecting wipes and barrier resistance of protective clothing. *JAMA Netw Open*. 2025;8(10):e2539307
doi:10.1001/jamanetworkopen.2025.39307

eMethods

eTable. Protective Clothing Models

eFigure. Microscopic Images of Fabrics at Low and High Magnification

This supplemental material has been provided by the authors to give readers additional information about their work.

© 2025 Kilinc-Balci FS et al. *JAMA Network Open*.

eMethods

Nine commonly used protective clothing (PC) models with various material compositions and constructions were selected to represent healthcare and laboratory settings (see eTable and eFigure). Liquid barrier resistance (LBR) (impact penetration [IP] by AATCC 42 and hydrostatic resistance [HR] by AATCC 127) was measured before wiping, immediately after, and after a four-hour dry period in an environmental chamber. Ten samples per PC model were tested per American National Standards Institute/American Association of Medical Instrumentation PB70 (AAMI hereafter).

PC were procured from the open market and subsequently cut and conditioned in our laboratories. The study included nine PC models, with ten units available per model for specimen preparation. Each unit was sampled once for each unique testing scenario, covering two test methods under five different conditions. Consequently, each unit of a given model yielded ten test specimens, corresponding to each unique testing scenario.

For example, the SMS-medium weight model A (Level 2, Medline) comprised 10 gown units. Each gown unit was carefully traced and labeled to ensure that the ten test specimens cut from it corresponded precisely to the following combinations:

- Impact Penetration (IP): Control, Wiped with QAA, Wiped with QA, Wiped and Dried with QAA, and Wiped and Dried with QA
- Hydrostatic Resistance (HR): Control, Wiped with QAA, Wiped with QA, Wiped and Dried with QAA, and Wiped and Dried with QA

Each specimen was uniquely labeled to prevent resampling of the same unit under identical conditions. This procedure was repeated for all remaining units across each PC model.

As a result, each model produced 50 data points for the IP test and 50 data points for the HR test. Overall, a total of 900 specimens were tested in this study.

Samples were conditioned for at least four hours before testing, following ASTM D1776, and tested immediately after gentle wiping on the fabric's outer surface. For drying conditions, samples were wiped, dried in an environmental chamber for four hours, and then tested.

eTable. Protective Clothing Models

AAMI PB70 Level	Protective Clothing Type	Manufacturer	Model Number	Fabric Type
Level 2	Isolation gown	Medline	NONLV200	SMS[(a)] - Medium weight (A)
	Isolation gown	Cardinal Health	AT4437-BD	SMS - Medium weight (B)
Level 3	Isolation gown	Halyard	54310	SMS - Heavy weight (A)
	Isolation gown	Medline	NONLV325	SMS - Heavy weight (B)
Level 4	Surgical gown	Medline	DYNJP2202	Poly-reinforced SMS
	Surgical gown	Halyard	92338	Spunbond-Film-SMS
Other	Coverall	DuPont	TY198TWHLG002500PI[(c)]	Flashspun
	Coverall	DuPont	TJ198TWHLG0025PI[(b)]	Coated flashspun

© 2025 Kilinc-Balci FS et al. *JAMA Network Open*.

	Isolation gown	Cardinal Health	7101PG	Laminated

[(a)] SMS: Spunbond-Meltblown-Spunbond
[(b)] Claimed hydrostatic resistance, American Association of Textile Chemists and Colorists (AATCC) 127: >254 cm
[(c)] Claimed hydrostatic resistance, AATCC 127: >121 cm

Statistical Analysis of HR (AATCC 127)

A total of 270 experiments were done to examine the effect of wiping on hydrostatic resistance. 180 were conducted on surgical and isolation gowns that claim conformance to AAMI. 30 were conducted on a gown model that does not claim conformance to AAMI, and 60 were conducted on two coverall models that are commonly used by emergency medical personnel and healthcare workers. Gowns that comply with AAMI classification standard include two level 2 isolation gown models, two level 3 isolation gown models, and two level 4 surgical gown models. The AATCC 127 (HR) was analyzed separately for gowns that claim conformance to AAMI and gowns and coveralls that do not as a function of wiping (no wiping, wipe type: QAA, and wipe type: QA) and fabric type. Analyses of variance (ANOVA) with Bonferonni adjusted, post-hoc, pair-wise comparisons were conducted for each condition. The p value reported in IBM SPSS is already adjusted for the number of post hoc comparisons made. The critical value for the Bonferonni adjusted p value is meant designed to be 0.05. However, for the purpose of this manuscript we used a critical value of ≤ 0.001 as the critical value. An additional condition of 'drying' after wiping (180 experiments) was omitted from the statistical model given that the main effect of allowing the fabric to dry after wiping was not found to be statistically significant ($p>0.05$).

Statistical Analysis of IP (AATCC 42)

A total of 270 experiments were done to examine the effect of wiping on impact resistance. 180 were conducted on surgical and isolation gowns that claim conformance to AAMI, 30 were conducted on a gown model that does not claim conformance to AAMI, and 60 were conducted on two coverall models that are commonly used by emergency medical personnel and healthcare workers. The spray IP (AATCC 42) was analyzed separately for gowns that claim conformance to AAMI and gowns and coveralls that do not as a function of wiping (no wiping, QAA wipe, and QA wipe) and fabric type. ANOVA with Bonferonni adjusted, post-hoc, pair-wise comparisons were conducted for each condition. The p value reported in IBM SPSS is already adjusted for the number of post hoc comparisons made. The critical value for the Bonferonni adjusted p value is meant designed to be 0.05. However, for the purpose of this manuscript we used a critical value of ≤ 0.001 as the critical value. An additional condition of 'drying' after wiping (180 experiments) was omitted from the statistical model given that the effect of allowing the fabric to dry after wiping was not found to be statistically significant ($p>0.05$).

Impact of Drying

The potential association between drying and LBR was examined by comparing the LBR (AATCC 127 and AATCC 42) of the fabrics that were not wiped and wiped and still wet with a new condition in which fabrics were wiped and then allowed to dry. 180 AATCC 127 and 180 AATCC 42 experiments were done by fabric type and wipe type to accommodate these comparisons. The AATCC 127 and AATCC 42 values after wiping and after wiping and drying were compared using multiple statistical tests (Wilcoxon rank sum, regressions, and robust and non-robust t-tests). Welch's t-test (unequal variances assumed) was used for the comparisons between IP or HR after wiping and IP or HR after wiping and drying and IP or HR before wiping and IP or HR after wiping and drying. The p values for a robust t test (non-equal variances assumed) in which wiping and drying were also compared to the no wipe condition with 0.001 significance.

© 2025 Kilinc-Balci FS et al. *JAMA Network Open*.

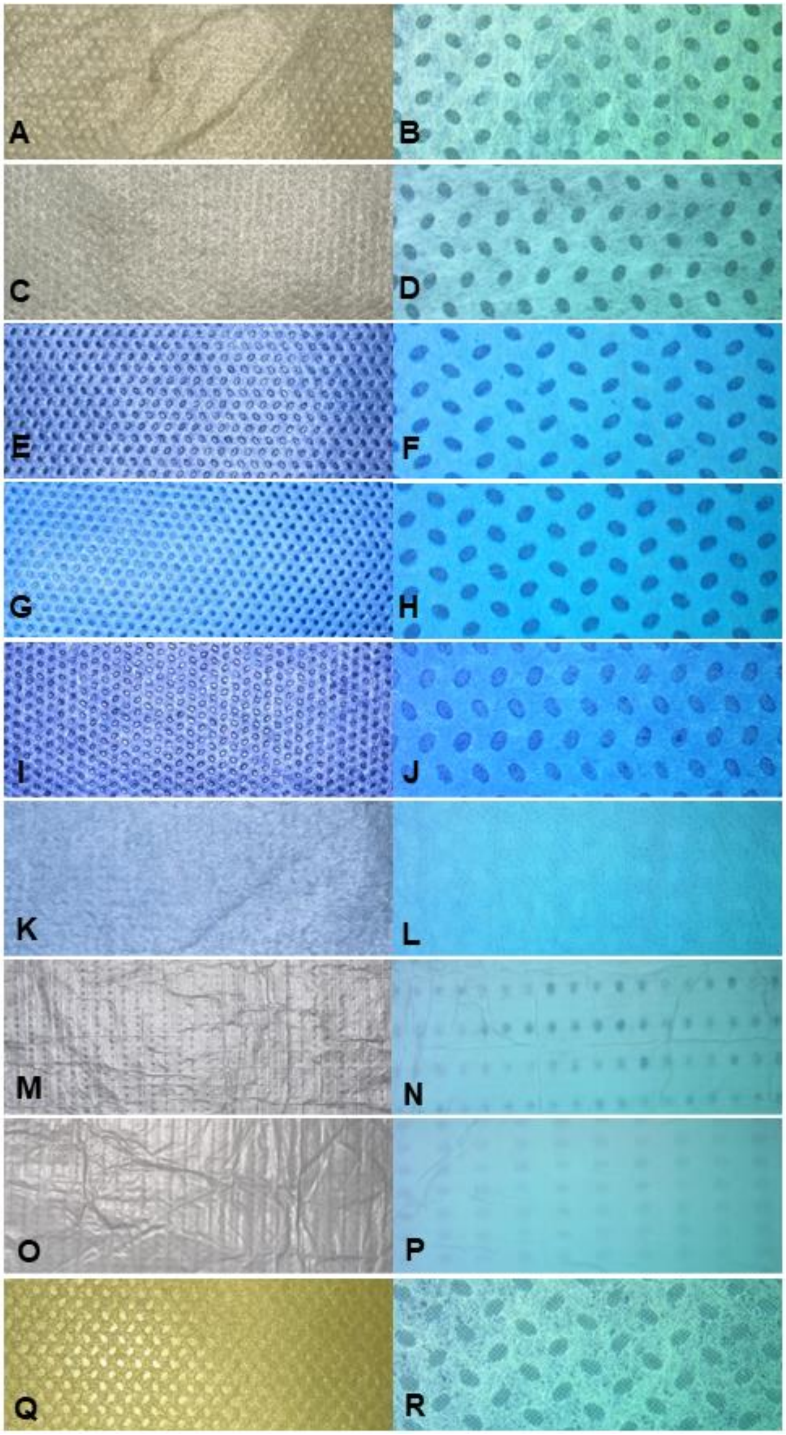

eFigure. Microscopic Images of Fabrics at Low and High Magnification

Note: Microscopic images of nine tested fabrics. For each fabric, the left panel shows the 2X magnification image and the right panel shows the 30X magnification image: (A, B): SMS - Medium weight (A); (C, D) :SMS - Medium weight (B); (E, F): SMS - Heavy weight (A); (G, H): SMS - Heavy weight (B); (I, J): Poly-reinforced SMS; (K, L): S-F-SMS; (M, N): Flashspun; (O, P): Coated Flashspun; (Q, R): Laminated.

© 2025 Kilinc-Balci FS et al. *JAMA Network Open*.
