# Supplementary material for: Disinfecting Wipes and Barrier Resistance of Protective Clothing
Source: JAMA Netw Open. 2025 Oct 24;8(10):e2539307. doi: 10.1001/jamanetworkopen.2025.39307 (PMC12552923; doi:10.1001/jamanetworkopen.2025.39307)
Supplement: Supplement 2. — Data Sharing Statement [file jamanetwopen-e2539307-s002.pdf]

Data Sharing Statement

Data
Data available: Yes
Data types: Data (not involving human participants)
How to access data: Data will be available to those who contact to the corresponding author.
When available: With publication

Supporting Documents
Document types: None

Additional Information
Who can access the data: anyone requesting the data
Types of analyses: for research purposes
Mechanisms of data availability: with a signed data access agreement
Any additional restrictions: NA
